# Downregulation of Cathepsin B expression alleviates periodontitis by reducing mitochondrial reactive oxygen species production and NOD-, LRR-, and pyrin domain-containing 3 -mediated pyroptosis

**DOI:** 10.3389/fimmu.2026.1762290

**Published:** 2026-03-06

**Authors:** Tianqi Wang, Xinran Liu, Jiaxin Li, Yan Ding, Yuan Yue, Jinle Li, Min Wang, Na Wei, Liang Hao

**Affiliations:** 1 State Key Laboratory of Oral Diseases & National Center for Stomatology & National Clinical Research Center for Oral Diseases, Department of Prosthodontics, West China Hospital of Stomatology, Sichuan University, Chengdu, China; 2 Division of Life Science and the Biotechnology Research Institute, Hong Kong University of Science and Technology, Clear Water Bay, Kowloon, Hong Kong SAR, China; 3 State Key Laboratory of Oral Diseases & National Center for Stomatology & National Clinical Research Center for Oral Diseases, Department of General Clinic, West China Hospital of Stomatology, Sichuan University, Chengdu, China; 4 State Key Laboratory of Oral Diseases & National Center for Stomatology & National Clinical Research Center for Oral Diseases, Department of Oral Implantology, West China Hospital of Stomatology, Sichuan University, Chengdu, China

**Keywords:** periodontitis, pyroptosis, Cathepsin B, NOD, LRR, domain-containing 3, reactive oxygen species

## Abstract

**Background:**

Periodontitis is one of the most common oral inflammatory diseases, and NOD-, LRR-, and pyrin domain-containing 3 (NLRP3)-mediated pyroptosis plays a crucial role in its pathogenesis. Cathepsin B (CTSB), a lysosomal cysteine protease, is closely associated with programmed cell death. Our study aimed to investigate the role of CTSB in periodontitis development through the NLRP3-mediated pyroptosis pathway and further explore the mechanism through which CTSB triggers NLRP3 activation.

**Methods:**

Ligature-induced periodontitis were established in BALB/c mice. Adeno-associated virus (AAV) was employed to downregulate CTSB expression in periodontal tissues. Small-interfering RNA (siRNA) was used to inhibit CTSB expression in macrophages for *in vitro* experiments. Micro-computed tomography (micro-CT) was employed to evaluate bone resorption. Immunohistochemistry, immunofluorescence, quantitative real-time polymerase chain reaction, western blotting, and enzyme-linked immunosorbent assay were used to examine CTSB expression, pyroptosis proteins, and inflammatory factors. MitoSOX Red and DCFH-DA staining were applied to detect mitochondrial and intracellular reactive oxygen species (ROS) levels.

**Results:**

CTSB downregulation significantly reduced alveolar bone resorption and macrophage infiltration in periodontitis. Although NLRP3 and inflammatory cytokine levels increased in periodontitis, they were effectively reduced after CTSB inhibition in the periodontal region. Consistent with *in vivo* experiments, CTSB knockdown in macrophages also suppressed pyroptosis. Furthermore, both mitochondrial and intracellular ROS levels were decreased after CTSB inhibition.

**Conclusions:**

Inhibiting CTSB expression alleviates periodontitis, primarily by suppressing NLRP3-mediated pyroptosis in macrophages. The mechanism through which CTSB activates NLRP3 likely involves inducing mitochondrial ROS generation. These findings reveal a novel mechanistic axis (CTSB-mitochondrial ROS-NLRP3) in periodontitis, highlighting a potential conceptual target for future therapeutic strategies.

## 1 Introduction

Periodontitis, a chronic inflammatory disease, is characterized by the progressive destruction of periodontal supporting tissues. In severe cases, it can lead to tooth loosening or even tooth loss (1). Approximately 12.5% of the global population suffers from severe periodontitis, which significantly impacts the quality of life, with incidence expected to rise in the coming years (2, 3). Periodontitis is primarily attributed to pathogen infection and dysbiosis of the oral microbiota, which induces an immune inflammatory response (4). Persistent immune stimulation promotes excessive production of pro-inflammatory mediators by immunocytes, such as macrophages, leading to matrix degradation and alveolar bone resorption (5). Traditional non-surgical periodontal therapy methods like scaling and root planing remove plaque and calculus but often lead to disease recurrence (6).

Pyroptosis is a pro-inflammatory form of programmed cell death triggered by stimuli such as bacterial infection. It is primarily mediated by inflammatory caspases(e.g., caspase-1, -4, -5, and -11) and executed by Gasdermin family proteins (particularly GSDMD), leading to pore formation in the plasma membrane, cell swelling, rupture, and the release of potent inflammatory mediators. These mediators recruit and activate immune cells, thereby amplifying the local inflammatory response (7). The canonical pathway is activated by inflammasomes, leading to caspase-1 activation. In contrast, the noncanonical pathway is directly triggered by cytosolic lipopolysaccharide (LPS), which activates human caspase-4/5 or murine caspase-11 (8, 9) Pyroptosis contributes to periodontitis pathogenesis, with the NOD-, LRR-, and pyrin domain-containing 3 (NLRP3) inflammasome playing an essential role (10, 11). NLRP3 deficiency alleviates alveolar bone resorption, highlighting the importance of its activation mechanism in disease progression (12). Although the exact mechanisms remain unclear, NLRP3 inflammasome activation occurs via multiple pathways, including potassium-dependent/-independent and lysosomal membrane permeabilization. Additionally, the release of oxidized mitochondrial DNA and mitochondrial reactive oxygen species (ROS) can also activate NLRP3 (11, 13).Therefore, NLRP3 antagonists targeting this activation process hold clinical potential.

Cathepsin B (CTSB), a lysosomal cysteine protease, is initially an inactive precursor. Under pathological stimuli, increased lysosomal membrane permeability enables CTSB maturation and release into the cytoplasm (14). CTSB contributes to pathogenesis in human diseases like neurological disorders, cancer, and pancreatitis (15–17) through mechanisms involving programmed cell death induction, cell proliferation regulation, extracellular matrix degradation, and mitochondrial oxidative stress induction (14, 16, 18). CTSB is highly expressed in fibroblasts from chronic periodontitis patients (19), with levels in gingival crevicular fluid correlating with disease progression (20). CTSB primarily originates from macrophages in periodontal tissues and promotes macrophages to release pro-inflammatory factors under pathological stimuli (21). Although its exact pathogenic mechanisms require further elucidation, these findings suggest a close association between CTSB and periodontitis.

CTSB triggers programmed cell death, particularly pyroptosis, in inflammatory diseases. Studies have shown that CTSB activates NLRP3 inflammasome-mediated pyroptosis in pancreatitis and diabetic cardiomyopathy (22, 23). A previous study found that CTSB could induce mitochondrial ROS generation (14). Given that mitochondrial ROS may activate NLRP3, we hypothesized that CTSB promotes periodontitis progression via mitochondrial ROS-induced NLRP3 inflammasome activation and pyroptosis.

## 2 Materials and methods

### 2.1 Animals

Eighty 4-week-old male BALB/c mice, housed in the specific pathogen-free facility, were used to establish a periodontitis model. The mice were divided into four groups at random(n = 20 per group) (1): control group without intervention (Control) (2); negative control group transfected with adeno-associated virus (AAV)-GFP-blank virus (Control + AAV-GFP) (3); periodontitis group transfected with AAV-GFP-blank virus (LIP+AAV-GFP) (4); periodontitis group transfected with AAV-CTSB virus (LIP+AAV-CTSB). All procedures were in compliance with the Institutional Animal Care and Use Committee (IACUC) guidelines of West China Hospital of Stomatology and approved by the Medical Ethics Committee of Sichuan University (Approval No.: WCHSIRB-D-2024-760).

### 2.2 AAV transfection

Following a 1-week adaptation, mice received kanamycin (J&K Scientific; 0.5 mg/ml in drinking water) for 3 days to eliminate infections, then sterile water for 3 days. Subsequently, AAV-CTSB (AAV‐U6‐CTSB‐shRNA‐CAG‐EGFP; GeneChem) was transfected in the mice periodontal region for 21 days to downregulate CTSB expression (24). The sequence for CTSB-shRNA is provided in **Supplementary Table 1**. Empty AAV vectors (abbreviated as AAV-GFP) served as negative controls. Viral particles were diluted in phosphate-buffered saline(PBS) to 2.5 × 10¹^0^ vg/mL, and 40 μL of the working solution was bilaterally injected into the buccal and lingual periodontal ligament of maxillary molars. Three evenly distributed injections per side were administered every 2 days.(8 total applications over 21 days) (25).

### 2.3 Periodontitis model establishment and sample harvest

Four weeks post-AAV transfection, mice were anesthetized by intraperitoneal injection of tribromoethanol (avertin, 1.25% solution) at a dose of 0.2 ml per 10g body weight. A 5-mm 5–0 silk ligature (knot-end buccal) was clipped bilaterally between the first and second maxillary molars. The knot remained buccal, with the remainder placed in the lingual gingival sulcus of maxillary first molars (26). On the 9th day, mice were euthanized by exposure to carbon dioxide (CO_2_) using a gradual fill method at a flow rate of 30-70% of the chamber volume per minute, followed by cervical dislocation for confirmation of death after successful induction of the periodontitis model (27, 28). For micro-CT and histological examination, right maxillary bones were fixed in 4% PFA (4 °C/overnight), water-rinsed (8h), and stored in 70% ethanol (4 °C). Left maxillary bones were divided for molecular analyses: RNA samples in RNAlater™ (Thermo Fisher Scientific,USA) at -80 °C; protein samples directly at -80 °C (29).

### 2.4 Micro-CT scanning

Right maxillary samples were scanned using the vivaCT 80 system (Scanco Medical, Switzerland) at 10 μm resolution. Three-dimensional (3D) models were reconstructed to analyze alveolar bone resorption areas, with cementoenamel junction (CEJ)-alveolar bone crest (ABC) distances measured in sagittal images.

### 2.5 Immunofluorescence staining and immunohistochemistry

After micro-CT scanning, samples were decalcified, paraffin-embedded, and sectioned(5 μm thickness). For immunofluorescence staining, primary antibodies—anti-F4/80 (1:100; Huabio, China), anti-CTSB (1:100;Proteintech,USA), and anti-NLRP3 (1:100; Huabio)—were used at a 1:100 dilution to incubate tissue sections. Secondary antibodies, goat anti-mouse Immunoglobulin G (IgG) (Dylight594) and goat anti-rat IgG (Dylight 488) were applied according to the manufacturer’s instructions (Abcam, UK). Nuclei were stained with 4′,6-diamidino-2-phenylindole (Cell Signaling Technology, USA) and observed by fluorescence microscopy. For immunohistochemical staining, the primary antibodies anti-F4/80 (1:100; Huabio) and anti-CTSB (1:100,Proteintech), along with the secondary antibody (Abcam) were used.

### 2.6 Cell culture, small interfering RNA transfection and inflammation model establishment

For *in vitro* experiments, RAW264.7 murine macrophages were cultured in high-glucose Dulbecco’s modified Eagle’s Medium (DMEM). At 80% confluence, viable cells were plated in six-well plates (2×10^5^ cells/well). After 24-hour (30%-40% confluence), transfection was performed with 1 μmol/L siRNA. ([CTSB] or [NC], Sangon Biotech,China). The transfection working solution was prepared using 250μL Opti-MEM (Gibco, USA), 3.5μL Lipofectamine™ 3000 (Thermo Fisher Scientific), and 2.5μL siRNA. Following 24-hour transfection, cells were stimulated with 1 μmol/L lipopolysaccharide (LPS,derived from Escherichia coli O55:B5) to establish an inflammatory model. Cell samples were then collected 24 hours post-LPS stimulation for subsequent analyses (30). Cell samples were allocated into six experimental groups (1): Control (2), Control + siRNA (NC) (3), Control + siRNA (CTSB) (4),LPS (5), LPS + siRNA (NC), and (6) LPS + siRNA (CTSB). siRNA sequences are listed in **Supplementary Table 2**.

### 2.7 Quantitative reverse transcription-polymerase chain reaction

Total RNA was extracted from cells and left maxillary bone samples using the RNApure Kit (Bioteke,China). Complementary DNA (cDNA) was generated with RevertAid Master Mix Kit (Thermo Fisher Scientific). cDNA was added in 384-well plates (Axygen^®^) along with PowerUp™ SYBR™ Green (Thermo Fisher Scientific) and primers for qRT-PCR. Primers are presented in **Supplementary Table 3**.

### 2.8 Protein extraction and western blotting

Proteins were extracted using Total Protein Extraction Kit (SAB, USA). Samples were mixed with 5× loading buffer, denatured(100 °C,10min) and separated by sodium dodecyl sulfate-polyacrylamide gel electrophoresis based on molecular weight differences. Proteins were transferred to polyvinylidene fluoride (PVDF) membranes, blocked was 5% milk(1 h, room temperature),and incubated overnight(4°C) with primary antibodies:β-actin (1:20000; Abclonal, China); CTSB (1:1000; Cell Signal Technology); NLRP3 (1:1000; Cell Signal Technology); pro-caspase-1 (1:1000;Cell Signal Technology); cleaved-caspase-1 (1:1000; Abmart, China); GSDMD (1:1000; Huabio).The PVDF membranes were washed the next day, incubated for 1 h with horseradish peroxidase-conjugated goat anti-rabbit IgG secondary antibody (1:5000;Abclonal). Band visualization was achieved using the ChemiDoc™ MP (Bio-Rad), with subsequent quantification via Image J.

### 2.9 Enzyme-linked immunosorbent assay

Right maxillary homogenates and cell supernatants were collected for quantification of the inflammatory cytokine Interleukin-1β (IL-1β). ELISA was performed using a kit (Elabscience,China) following manufacturer’s protocols.

### 2.10 Measurements of mitochondrial ROS and intracellular ROS

Mitochondrial ROS was detected using MitoSOX™ Red Mitochondrial Superoxide Indicator (Yeasen; China). Intracellular ROS was measured with 2’-7’-dichlorodihydrofluorescein diacetate (DCFH-DA) probe (Beyotime ROS Assay Kit, China). Cells were washed with PBS, stained with Hoechst 33258 (Servicebio, China) for nuclei visualization, and imaged under a fluorescence microscope (Leica DMI8, Germany). All procedures were performed in the dark (31).

### 2.11 Statistical analysis

Data analysis used GraphPad Prism 9.5 with a significance level of p < 0.05. Experimental results were presented as means ± standard deviation from≥3 independent experiments. Parametric data (bone resorption, mRNA/protein expression levels) used t-tests (two groups) or one-way Analysis of Variance with Dunnett’s *post hoc* test (multi-group). Non-parametric data (positive cell percentages) used Kruskal-Wallis test, followed by appropriate non-parametric *post hoc* analyses.

## 3 Results

### 3.1 Downregulation of CTSB expression alleviates alveolar bone destruction in periodontitis

**Figure 1:**
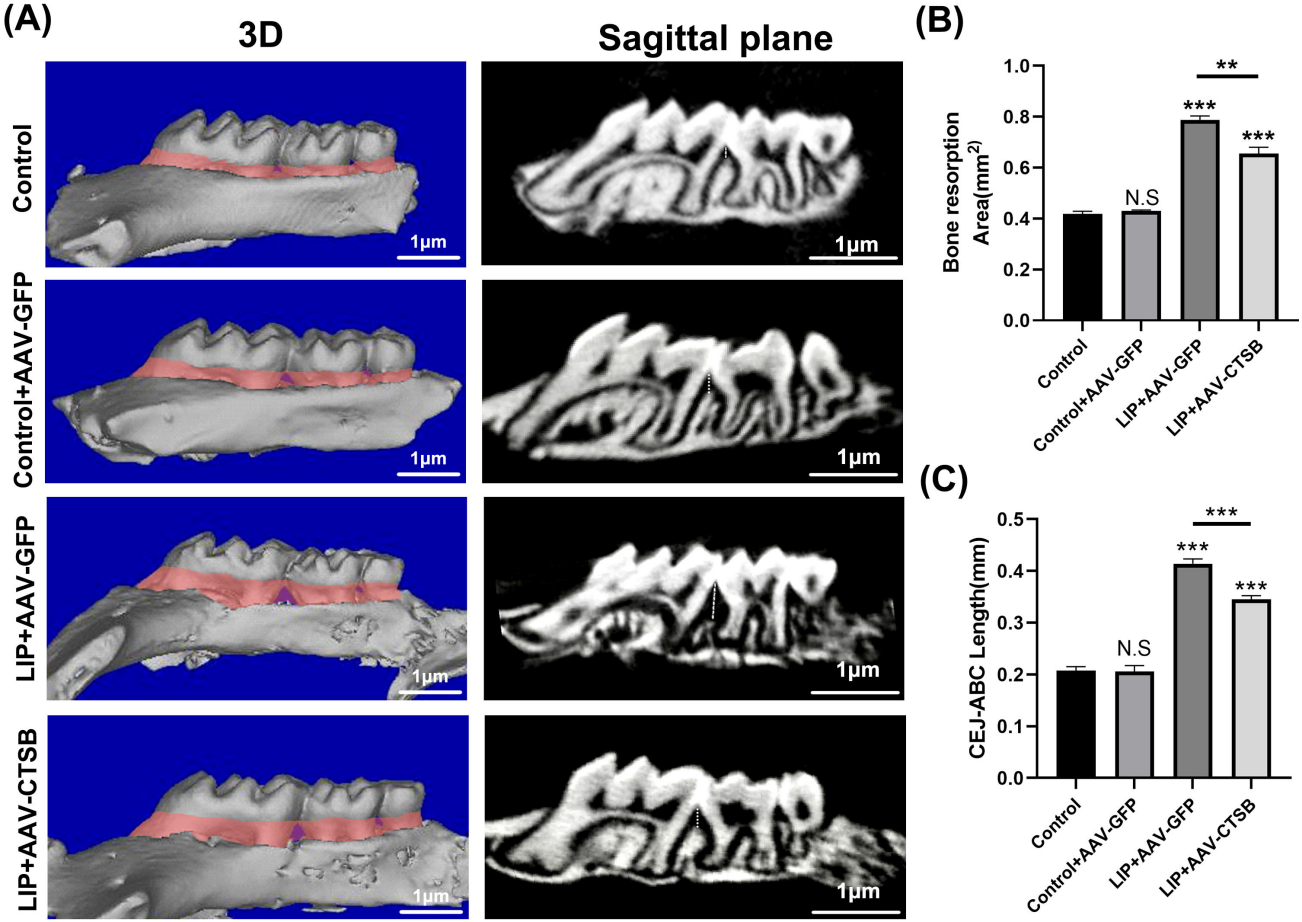
AAV-CTSB alleviates alveolar bone destruction in mice periodontitis. **(A)** Three-dimensional reconstruction images and sagittal plane images of the maxillary bone categorized by group. The red shaded area represents the amount of alveolar bone resorption, and the white dotted line represents the distance from the cementoenamel junction (CEJ) to the alveolar bone crest (ABC). Quantitative analysis of **(B)** the bone resorption area and **(C)** distance from CEJ to ABC. NS, not significant, **p<.01, ***p<.001.

Micro-CT scanning showed significant alveolar bone loss as both bone resorption volume and the CEJ-ABC distance were greater in the LIP + AAV-GFP group than in the Control + AAV-GFP group (**Figures 1A–C**). These results confirmed that the ligature-induced periodontitis model has been successfully established. The LIP + AAV-CTSB group exhibited reduced bone resorption and a shorter CEJ-ABC distance than the LIP + AAV-GFP group (**Figures 1A–C**), suggesting CTSB downregulation protects against periodontitis-induced alveolar bone loss.

### 3.2 Downregulation of CTSB expression leads to a reduction of macrophage infiltration

**Figure 2:**
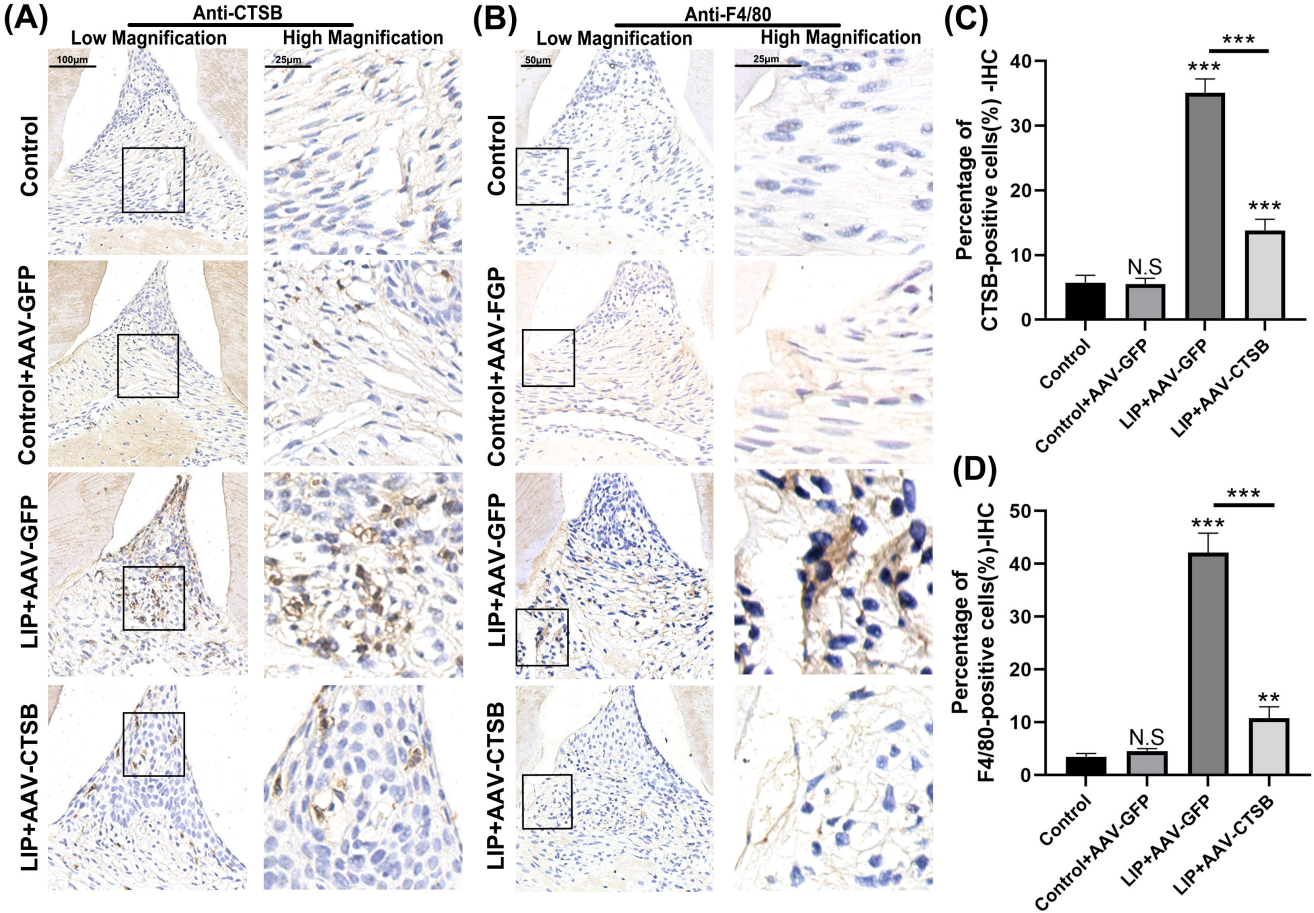
AAV-CTSB reduces macrophage infiltration in periodontal lesions. **(A)** Immunohistochemistry (IHC) analysis of CTSB in the periodontal area of each group. **(B)** IHC staining of F4/80 in the periodontal area of each group. **(C)** Quantitative analysis of CTSB-positive cells. **(D)** Quantification of F4/80-positive cells in each group. NS, not significant, **p<.01, ***p<.001.

AAV was used to inhibit CTSB expression in the periodontal region, as confirmed by IHC (**Figure 2A**). To further explore CTSB pathogenesis, we assessed macrophage infiltration by observing the expression of the surface marker F4/80 on macrophages via IHC (**Figure 2B**). IHC results revealed significantly decreased CTSB-positive cells in the LIP + AAV-CTSB group compared to the LIP + AAV-GFP group, indicating that AAV transfection successfully reduced CTSB expression. No significant difference was observed between the Control + AAV-GFP group and the Control group, suggesting that AAV-GFP transfection did not influence CTSB expression levels (**Figure 2C**). F4/80-positive cells significantly increased in the LIP + AAV-GFP group compared with the Control + AAV-GFP and Control groups, but decreased in the LIP + AAV-CTSB group compared to the LIP + AAV-GFP group (**Figure 2D**). These results suggest that enhanced macrophage infiltration contributes to periodontitis and that this increase could be mitigated by the downregulation of CTSB expression.

### 3.3 Downregulation of CTSB expression decreases NLRP3-mediated pyroptosis in macrophages and reduces the production of inflammatory cytokines

**Figure 3:**
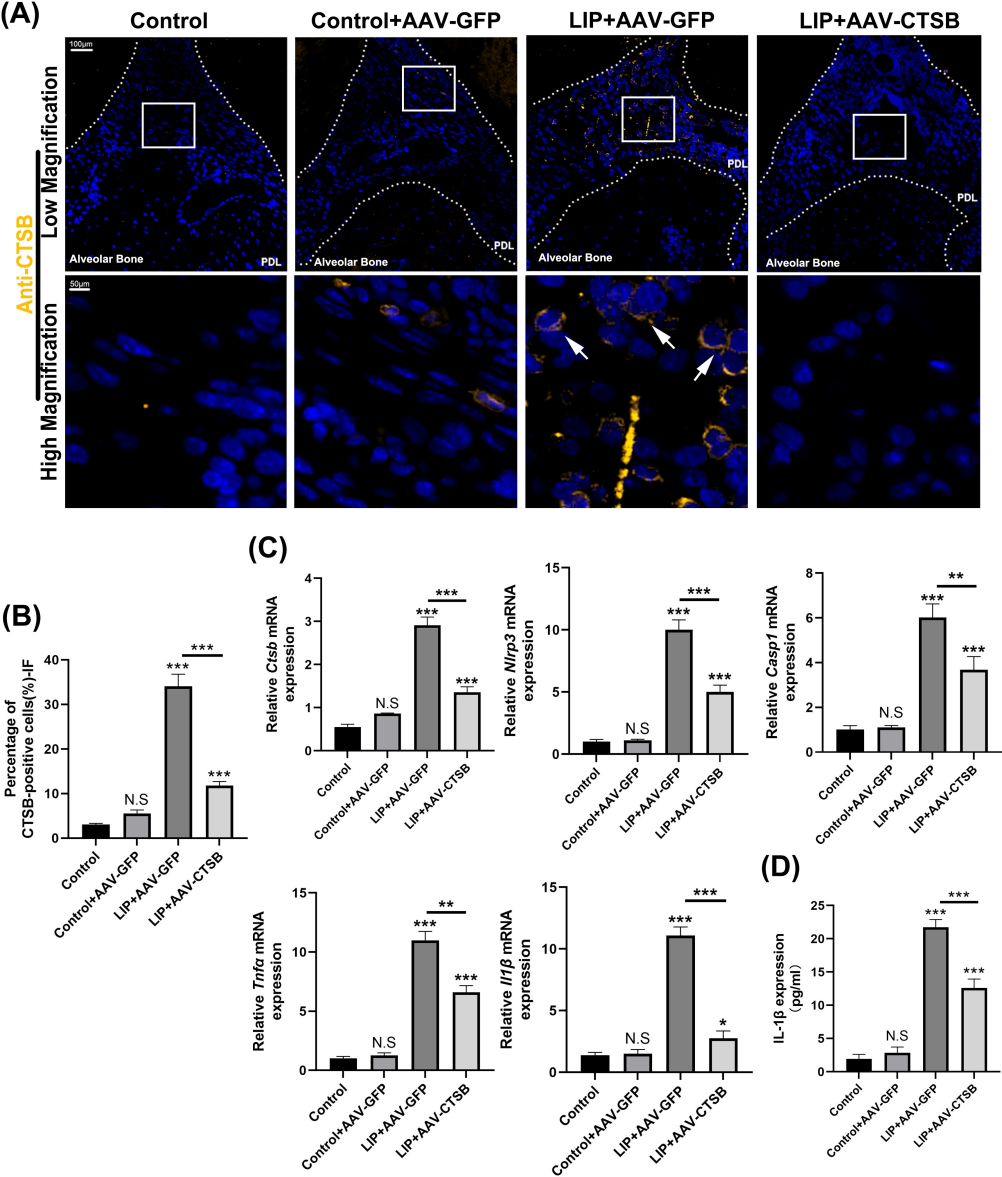
AAV-CTSB inhibits NLRP3-mediated pyroptosis and the production of inflammatory factors in mice periodontitis. **(A)** Immunofluorescence (IF) staining of CTSB in the periodontal tissues of each group. **(B)** Quantification of CTSB-positive cells in each group. **(C)** Relative mRNA expression levels of *Ctsb, Nlrp3, Casp1, Tnfα, and Il1β* in the periodontal tissues of each group as detected by quantitative real-time polymerase chain reaction. **(D)** Interleukin-1β concentration as detected by enzyme-linked immunosorbent assay. NS, not significant, *p<.05, **p<.01, ***p<.001..

**Figure 4:**
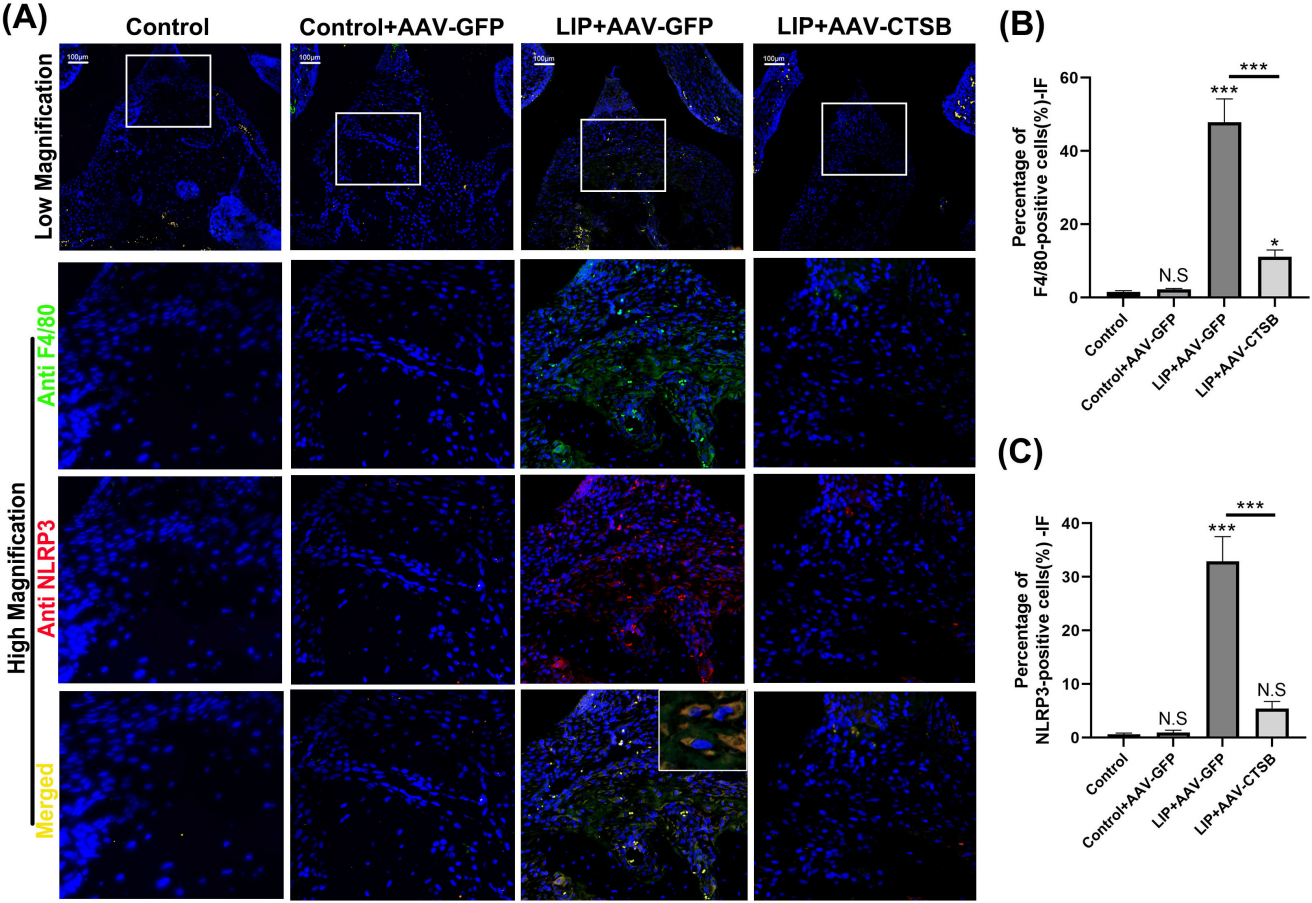
AAV-CTSB decreases NLRP3-mediated pyroptosis in mice macrophages. **(A)** Immunofluorescence staining of F4/80 and NLRP3 in the periodontal tissues of each group. **(B)** Quantification of F4/80-positive cells in each group. **(C)** Quantitative analysis of NLRP3-positive cells. NS, not significant, *p<.05, **p<.01, ***p<.001.

AAV transfection successfully downregulated CTSB expression, which was also verified by IF staining (**Figures 3A, B**). qRT-PCR analysis revealed the highest relative mRNA levels of NLRP3 in the LIP + AAV-GFP group with marked reduction in the LIP + AAV-CTSB group (**Figure 3C**). *Tumor necrosis factor-α*(*Tnf-α*)and *Il-1β* mRNA levels significantly increased in the LIP + AAV-GFP group but decreased with CTSB suppression (**Figure 3C**). ELISA results were consistent with qRT-PCR findings, demonstrating an elevated IL-1β production in the LIP + AAV-GFP group and a decrease in the LIP + AAV-CTSB group (**Figure 3D**). Pyroptosis, which mainly occurs in macrophages, is crucial for periodontitis development. Our previous results showed that CTSB downregulation reduces macrophage infiltration. Based on previous findings demonstrating the capacity of CTSB to induce NLRP3-mediated pyroptosis, we proposed CTSB promotes periodontitis by inducing macrophage pyroptosis. Therefore, we quantified the expression of F4/80 and NLRP3 in periodontal tissues. IF staining results showed a high degree of overlap between NLRP3-positive and F4/80-positive cells, indicating that NLRP3-mediated pyroptosis primarily occurs in macrophages (**Figure 4A**). Notably, both cell types exhibited the highest proportion in the periodontitis group but decreased significantly with CTSB inhibition (**Figures 4B, C**). These results confirmed our hypothesis that CTSB suppression alleviates periodontitis by inhibiting NLRP3-mediated pyroptosis.

### 3.4 Downregulation of CTSB expression alleviates NLRP3-mediated pyroptosis by reducing mitochondrial ROS production

**Figure 5:**
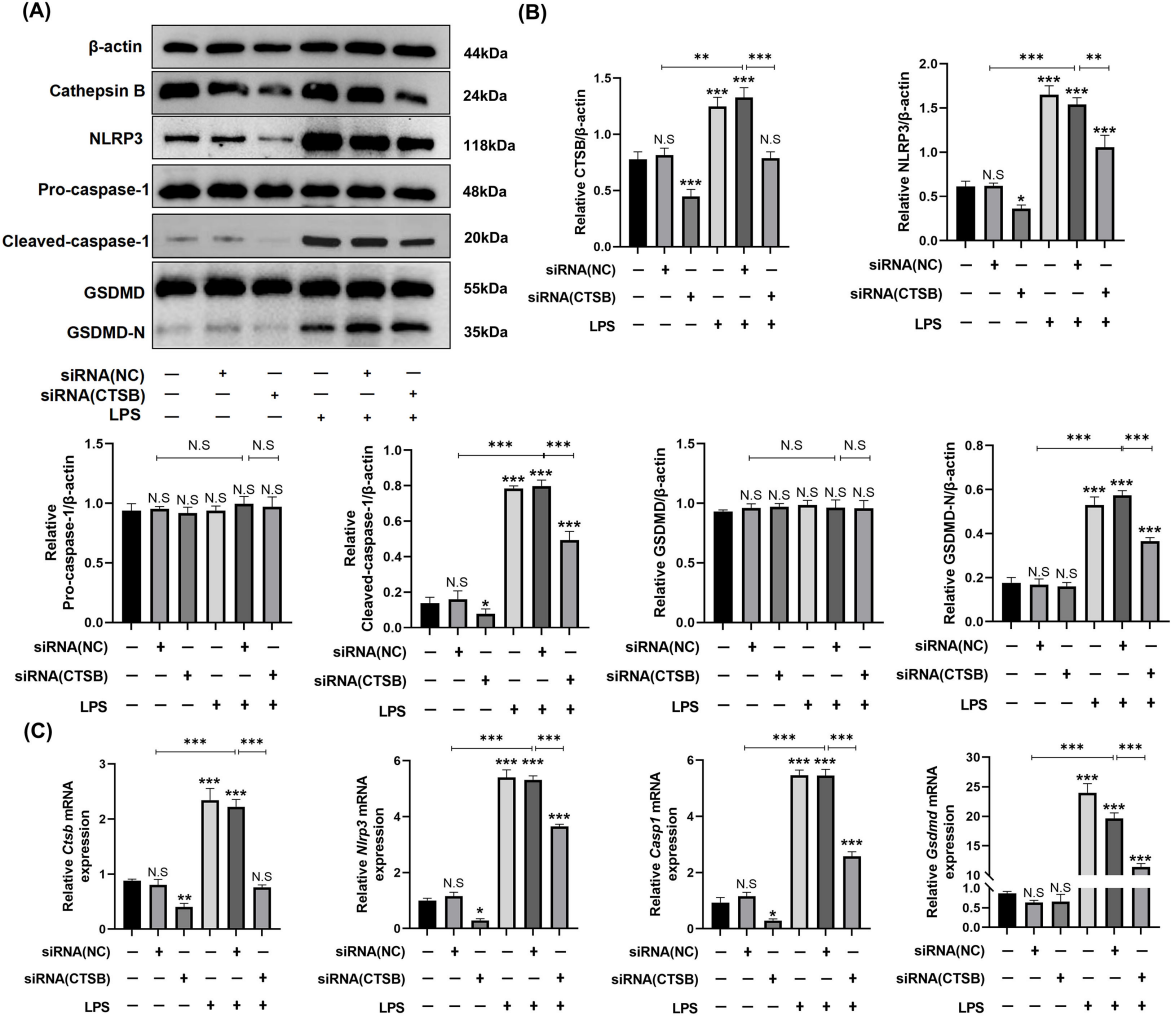
AAV-CTSB alleviates NLRP3-mediated pyroptosis in *in vitro* experiments. **(A, B)** western blotting and quantification analysis of CTSB, NLRP3, pro-caspase-1, cleaved-caspase-1, gasdermin-D (GSDMD), and GSDMD-N expression in the macrophage cell lines of each group. **(C)** Relative mRNA expression levels of *Ctsb, Nlrp3, Casp1, and Gsdmd* in macrophages as detected by quantitative real-time polymerase chain reaction. NS, not significant, *p<.05, **p<.01, ***p<.001.

Based on our prior findings that pyroptosis primarily occurs in macrophages, we cultured RAW264.7 macrophages *in vitro* to further explore CTSB’s role in initiating NLRP3-mediated pyroptosis and to elucidate the mechanisms by which CTSB activates NLRP3. We used siRNA transfection to downregulate CTSB gene expression in the cells. Subsequent western blotting and qRT-PCR analyses showed a decrease in CTSB expression levels in the Control + siRNA (CTSB) group compared to the control group, indicating that siRNA successfully inhibited CTSB expression. Western blotting results showed upregulated expression levels of NLRP3 inflammasome and pyroptosis execution markers, including cleaved caspase-1 and GSDMD-N, in both the LPS and the LPS + siRNA (NC) groups. In contrast, these proteins were markedly downregulated in the LPS + siRNA (CTSB) group, suggesting that CTSB inhibition effectively suppressed NLRP3-mediated macrophage pyroptosis (**Figures 5A, B**). The qRT-PCR results were consistent with the changes in protein levels observed in the western blotting analysis (**Figure 5C**).

**Figure 6:**
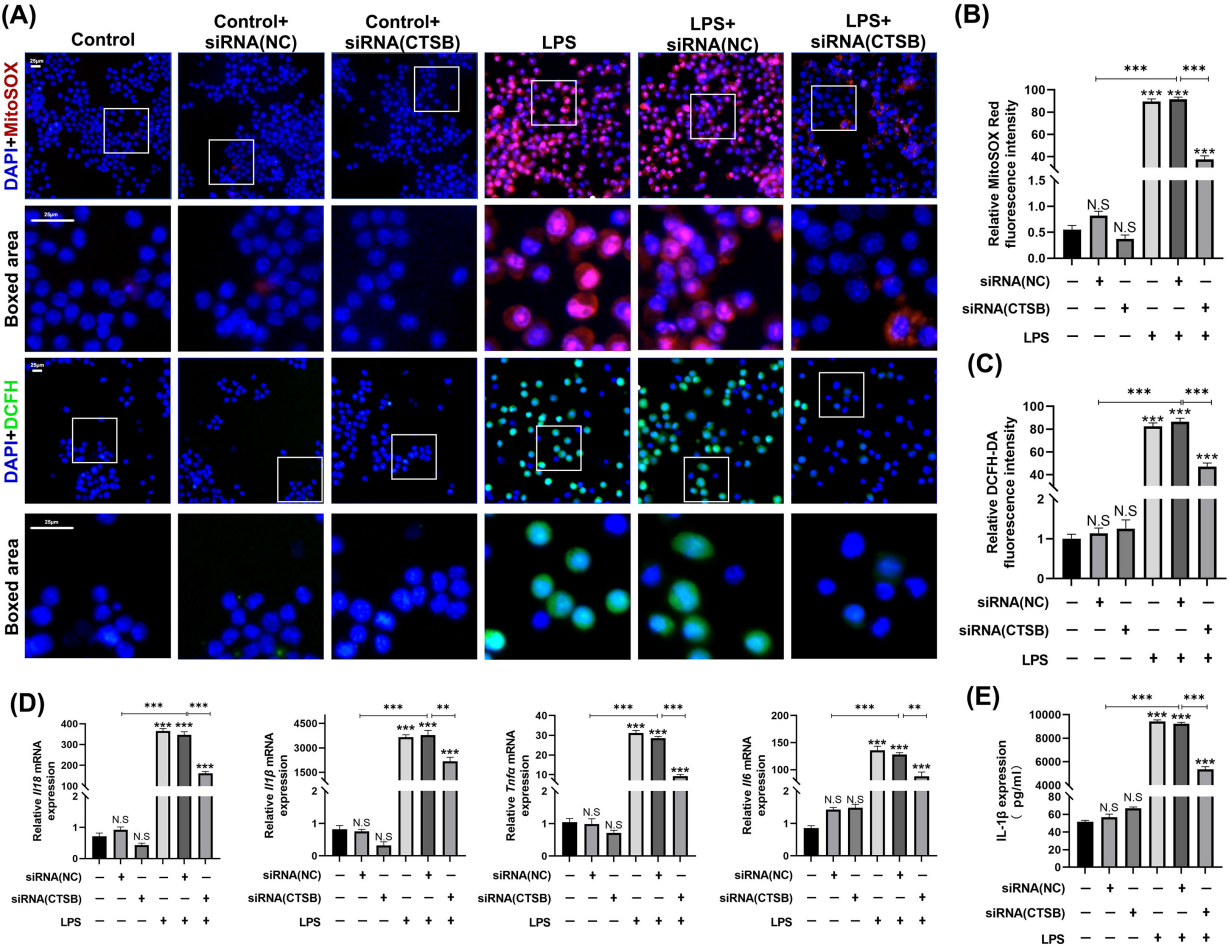
AAV-CTSB reduces mitochondrial reactive oxygen species (ROS) production and pyroptosis downstream inflammatory factors. **(A)** MitoSOX and 2’-7’-dichlorodihydrofluorescein diacetate (DCFH-DA) staining applied to detect mitochondrial and intracellular ROS production. **(B)** Quantitative analysis of relative MitoSOX red fluorescence intensity in each group. **(C)** Quantitative analysis of relative DCFH-DA fluorescence intensity in each group. **(D)** Relative mRNA expression levels of *Il18, Il1β, Tnfα, and Il6* in macrophages as detected by quantitative real-time polymerase chain reaction. **(E)** The expression level of interleukin-1β in the cell supernatant of different groups as detected by enzyme-linked immunosorbent assay. NS, not significant, **p<.01, ***p<.001.

To assess mitochondrial ROS production and intracellular ROS levels, we performed MitoSOX and DCFH-DA staining (**Figure 6A**). Findings indicated that both mitochondrial ROS production and intracellular ROS levels were markedly elevated in the LPS and LPS + siRNA (NC) groups. Conversely, these two parameters were reduced in the LPS + siRNA (CTSB) group. Thus, CTSB inhibition appears to mitigate mitochondrial ROS generation and its subsequent release into the cytoplasm (**Figures 6B, C**).

Furthermore, qRT-PCR analysis revealed a significant upregulation of pyroptosis downstream inflammatory cytokines *Il-1β* and *Interleukin-18 (Il-18)* in the LPS + siRNA (NC) group compared with the control + siRNA (NC) group while CTSB inhibition markedly reduced these cytokine levels. *Tnf-α* and *Interleukin-6 (Il-6)* exhibited similar expression patterns across the six groups (**Figure 6D**). Consistently, ELISA analysis of IL-1β expression levels in cell supernatants corroborated the findings from the qRT-PCR results (**Figure 6E**).

## 4 Discussion

In this study, we demonstrated the involvement of CTSB in periodontitis pathogenesis *in vivo* by activating NLRP3-mediated pyroptosis. This was further investigated using a macrophage cell line, uncovering the mechanism by which revealed that CTSB activates NLRP3 by inducing mitochondrial ROS production.

Periodontitis is a chronic inflammatory disease characterized clinically by progressive alveolar bone loss and attachment loss. These manifestations stem from dysregulated host immune responses to pathogenic biofilms, leading to extensive immune cell infiltration that exacerbates tissue destruction (32). Pyroptosis, a pro-inflammatory form of programmed cell death triggered by microbial infection or inflammatory stimuli, has been reported to contribute to the pathogenesis of periodontitis (11). Pyroptosis amplifies inflammation through NLRP3 inflammasome activation, gasdermin D (GSDMD)-mediated pore formation, and release of inflammatory factors IL-1β and IL-18 (33). Our experimental data showed an increase of NLRP3 expression in periodontitis group *in vivo* (**Figures 3**, **4**) and LPS groups *in vitro* (**Figure 5**). Pyroptosis executive markers (**Figure 5**) and its downstream inflammatory cytokines (**Figures 3**, **6**) were also elevated, aligning with the its established role in exacerbating periodontal inflammation. In this study, we employed a multi-layered approach to assess pyroptosis. In addition to detecting the upstream regulator NLRP3 and the downstream effector Il-1β, we confirmed the critical execution event—cleavage of caspase-1 and GSDMD-N—in our *in vitro* experiments (**Figure 5**), which aligns with the *in vivo* findings. These findings suggest that NLRP3-mediated pyroptosis is significantly involved in periodontitis pathogenesis consistent with previous studies (10, 34). Macrophages serve as pivotal immune regulators in periodontitis, orchestrating pathogen clearance, cytokine production, and antigen presentation (35). Our immunofluorescence results revealed co-localization of NLRP3 and the macrophage marker F4/80 in periodontal tissues (**Figure 4**), demonstrating that NLRP3-mediated pyroptosis occurs predominantly in macrophages within the periodontal lesion. This prompts our *in vitro* validation using RAW264.7 macrophages.

NLRP3 inflammasome activation is essential for pyroptosis, facilitating caspase-1 activation, which leads to proteolytic processing and release of IL-1β and IL-18 cytokines, thereby establishing inflammatory microenvironment (7, 36). Elucidating NLRP3 activation mechanisms is critical, as targeting this process could reduce inflammation-induced alveolar bone resorption in periodontitis patients and provide a molecular basis for novel therapeutics (12). NLRP3 activation likely involves multiple pathways, including mitochondrial ROS production, lysosomal disruption, and ion channel alteration (37). Critically, our study identified significantly elevated mitochondrial and intracellular ROS production under LPS stimulation, suggesting that mitochondria‐derived ROS contributes substantially to NLRP3 activation (**Figure 6**).

Cathepsin B (CTSB), a cysteine protease, is localized in lysosomes and expressed in most cell types. Physiologically, CTSB exists in its precursor form within lysosomes, facilitating intracellular protein turnover and regulating cell proliferation (16). Pathogen-induced lysosomal destabilization triggers CTSB release into the cytoplasm, activating cell death pathways and driving pathogenesis in cancer, neurological, and cardiovascular disorders (15, 16, 18). Although COX et al. identified high CTSB expression in periodontitis, its specific pathogenic role in the disease remains underexplored (19). In our study, we downregulated CTSB expression using AAVs and observed a reduction in bone resorption and macrophage infiltration in the LIP + AAV-CTSB group compared to the LIP + AAV-GFP group (**Figures 1**, **2**). *In vitro*, downregulating CTSB expression in macrophages attenuated pro-inflammatory cytokines secretion. (**Figure 6**). These results collectively demonstrate that CTSB serves as a crucial mediator in the pathogenesis and progression of periodontitis, and inhibiting CTSB expression could ameliorate inflammation.

:e12586Evidence suggests that the leakage of lysosomal CTSB into the cytosol contributes to NLRP3 inflammasome activation, leading to pyroptosis in the progression of certain inflammatory diseases such as acute pancreatitis, diabetic cardiomyopathy, and osteoarthritis (22, 23, 38). In our study, inhibiting CTSB expression led to the downregulation of NLRP3 expression in both *in vivo* and *in vitro* experiments, as shown in **Figures 4**, **5**. Concurrently, the expression of downstream pyroptosis-related proteins also decreased. Our study is the first to elucidate the pathogenic role of this pathway in periodontitis. Under pathological conditions, the leakage of CTSB from lysosomes facilitates the formation of its mature form (24 kDa, **Figure 4**), which plays a pivotal role in the decline of mitochondrial membrane potential, thereby resulting in the production of mitochondrial ROS (39).This results aligns with the previous study confirming that CTSB is responsible for the increase in mitochondrial ROS generation and pro-inflammatory cytokines, leading to memory impairment (40). As discussed above, mitochondria‐derived ROS generation activates NLRP3 inflammasome. Our findings revealed that CTSB inhibition significantly reduced mitochondrial and intracellular ROS levels (**Figure 6**), demonstrating that CTSB promotes NLRP3 inflammasome activation through inducing mitochondrial ROS generation. Based on these findings regarding the relationship between pyroptosis and periodontitis, as well as the role of CTSB in pyroptosis, targeting this CTSB-mitochondrial ROS-NLRP3 axis could mitigate inflammation-induced tissue damage in periodontitis and inhibition of CTSB expression presents a potential therapeutic strategy for attenuating inflammatory responses in periodontitis.

Our study elucidates that the CTSB-mitochondrial ROS-NLRP3 pyroptosis axis drives periodontitis progression, thereby nominating it as a potential target for intervention. However, translating this finding into therapy faces significant challenges. Cathepsin B plays crucial physiological roles in lysosomal proteolysis and cellular turnover (41). Systemic, long-term inhibition may therefore carry unforeseen risks. Currently, most CTSB inhibitors (e.g., CA-074) are broad-spectrum tools used in preclinical research, lacking the specificity and safety profiles for human clinical application, especially in a chronic inflammatory context like periodontitis (42–44). Future efforts should focus on developing localized delivery systems (e.g., sustained-release hydrogels or tissue-targeted nanoparticles) to achieve site-specific modulation within the periodontal pocket, thereby minimizing systemic exposure (45, 46). Furthermore, validating the efficacy and safety of inhibiting this axis in advanced disease models is a necessary precursor to any therapeutic development. Thus, our work primarily provides a novel mechanistic framework; its therapeutic value will depend on future innovations in targeted drug delivery and inhibitor design.

This study has some limitations. First, the murine model may not fully replicate human periodontal pathology, and this study used only male mice to control for hormonal variability. Future studies should include both sexes to evaluate potential differences, though the core NLRP3-pyroptosis axis is conserved. Second, We acknowledge that using only the RAW264.7 cell line for *in vitro* experiments, although well-validated for pyroptosis research, is a limitation as immortalized lines differ from primary macrophages. Confirmation in primary cells like BMDMs is needed for future translational work. Third, AAV-mediated CTSB knockdown could theoretically affect non-macrophage cell types in the periodontal tissue. While the contribution of pyroptosis in cells like fibroblasts is likely minimal, a synergistic role cannot be ruled out. Thus, future studies employing macrophage-specific knockout models are needed to definitively validate the cellular specificity of this mechanism. Additionally, while the DCFH-DA probe indicated increased general oxidative stress, its relatively nonspecific nature for detecting distinct ROS species is noted (47, 48). Future studies employing more specific probes or alternative assays would help to further delineate the precise ROS profile involved.

In conclusion, CTSB drives periodontitis progression mainly by inducing mitochondrial ROS to activate NLRP3-mediated pyroptosis in macrophages. This mechanistic insight reveals the CTSB-mitochondrial ROS-NLRP3 axis as a novel conceptual target, informing future research into precise therapeutic strategies for periodontitis management. Future studies should explore the long-term effects of CTSB inhibition on periodontitis progression, validate findings in human clinical samples, and investigate additional molecular pathways involved in NLRP3 activation.

## Data Availability

The original contributions presented in the study are included in the article/**Supplementary Material**. Further inquiries can be directed to the corresponding authors.
