# Supplementary material for: Downregulation of Cathepsin B expression alleviates periodontitis by reducing mitochondrial reactive oxygen species production and NOD-, LRR-, and pyrin domain-containing 3 -mediated pyroptosis
Source: Front Immunol. 2026 Mar 6;17:1762290. doi: 10.3389/fimmu.2026.1762290 (PMC13002364; doi:10.3389/fimmu.2026.1762290)
Supplement: Supplementary file 1 [file DataSheet1.docx]

| **NO.** | **5’** | **STEM** | **Loop** | **STEM** | **3’** |
| --- | --- | --- | --- | --- | --- |
| Ctsb-RNAi(127826-1)-a | ACCGG | GACTTACAAATCAGGAGTATA | TTCAAGAGA | TATACTCCTGATTTGTAAGTC | TTTTT |
| Ctsb-RNAi(127826-1)-b | TCTAAAAAA | GACTTACAAATCAGGAGTATA | TCTCTTGAA | TATACTCCTGATTTGTAAGTC | C |

**Supplementary Table 1** AAV-CTSB-shRNA sequences

| siRNA | sense(5′–3′) | anti-sense (5′–3′) |
| --- | --- | --- |
| negative control | UUCUCCGAACGUGUCACGUTT | ACGUGACACGUUCGGAGAATT |
| mCathepsin B-1086 | UGGCAUUGAAUCAGAAAUUTT | AAUUUCUGAUUCAAUGCCATT |

**Supplementary Table 2** siRNAs sequences

| Gene | Forward primer (5′–3′) | Reverse primer (5′–3′) |
| --- | --- | --- |
| *Gaphd* | AGGTTGTCTCCTGCGACTTCA | CCAGGAAATGAGCTTGACAAA |
| *Ctsb* | GGCTGGACGCAACTTCTACAATG | TATGTCCTCACCGAACGCAACC |
| *Nlrp3* | CCAGACCTCCAAGACCACTACG | CAGAGAAGAGATGCTCCTCAATGC |
| *Casp-1* | AATACAACCACTCGTACACGTCTTG | ATCCTCCAGCAGCAACTTCATTTC |
| *Gsdmd* | ACTGAGGTCCACAGCCAAGAGG | GCCACTCGGAATGCCAGGATG |
| *Il-1β* | CAACCAACAAGTGATATTCTCCATG | ATCCACACTCTCCAGCTGCA |
| *Il-18* | CAAAGTGCCAGTGAACCCCAGAC | ACAGAGAGGGTCACAGCCAGTC |
| *Tnf-α* | AGGGTCTGGGCCATAGAACT | CCACCACGCTCTTCTGTCTAC |
| *Il-6* | CTCTGCAAGAGACTTCCATCCAGT | GAAGTAGGGAAGGCCGTGG |

**Supplementary Table 3** primers used for qRT-PCR
